# Structure and Properties of Poly(ethylene terephthalate) Fiber Webs Prepared via Laser-Electrospinning and Subsequent Annealing Processes

**DOI:** 10.3390/ma13245783

**Published:** 2020-12-18

**Authors:** Tomoki Tokuda, Ryo Tsuruda, Takuya Hara, Haruki Kobayashi, Katsufumi Tanaka, Wataru Takarada, Takeshi Kikutani, Juan P. Hinestroza, Joselito M. Razal, Midori Takasaki

**Affiliations:** 1 Department of Macromolecular Science and Engineering, Graduate School of Science and Technology, Kyoto Institute of Technology, Matsugasaki, Sakyo-ku, Kyoto 606-8585, Japan; tmk.edhf@gmail.com (T.T.); alterguitarist23@gmail.com (R.T.); karuata.bb.0124@gmail.com (T.H.); haruki@kit.ac.jp (H.K.); ktanaka@kit.ac.jp (K.T.); 2 Department of Materials Science and Engineering, Tokyo Institute of Technology, 2-12-1 Ookayama, Meguro-ku, Tokyo 152-8550, Japan; takarada.w.aa@m.titech.ac.jp; 3 School of Materials and Chemical Technology, Tokyo Institute of Technology, 4259-J3-142, Nagatsuta-cho, Midori-ku, Yokohama, Kanagawa 226-8503, Japan; kikutani.t.aa@m.titech.ac.jp; 4 Department of Fiber Science and Apparel Design, Cornell University, 135 Human Ecology Building, Ithaca, NY 14850, USA; jh433@cornell.edu; 5 Institute for Frontier Materials, Deakin University, Geelong, VIC 3216, Australia; joselito.razal@deakin.edu.au

**Keywords:** poly(ethylene terephthalate), melt-electrospinning, ultra-fine fibers, birefringence, crystallinity

## Abstract

Melt-electrospinning is an eco-friendly method for producing ultra-fine fibers without using any solvent. We prepared webs of poly(ethylene terephthalate) (PET) through melt-electrospinning using CO_2_ laser irradiation for heating. The PET webs comprised ultra-fine fibers of uniform diameter (average fiber diameter = 1.66 μm, coefficient of variation = 19%). The co-existence of fibers with high and low molecular orientation was confirmed through birefringence measurements. Although the level of high orientation corresponded to that of commercial highly oriented yarn, crystalline diffraction was not observed in the wide-angle X-ray diffraction (WAXD) analysis of the webs. The crystallinity of the webs was estimated using differential scanning calorimetry (DSC). The fibers with higher birefringence did not exhibit any cold crystallization peak. After annealing the web at 116 °C for 5 min, a further increase in the birefringence of the fibers with higher orientation was observed. The WAXD results revealed that the annealed webs showed crystalline diffraction peaks with the orientation of the c-axis along the fiber axis. In summary, the formation of fibers with a unique non-crystalline structure with extremely high orientation was confirmed.

## 1. Introduction

Non-woven fabrics have been attracting attention because of their applicability in a wide variety of end uses, such as filtration media, battery separators, packaging sheets, and medical materials. Spun bonding, melt blowing, and electrospinning processes are mainly used for manufacturing non-woven fabrics. Among these techniques, the electrospinning process, a spinning method for producing nonwovens with submicron- and nano-scale fibers, has been actively investigated in recent years [1,2,3,4,5,6,7,8,9,10,11,12,13,14,15]. The electrospinning method can be classified into two different processes: solution spinning and melt spinning. While solution-electrospinning requires a solvent, melt-electrospinning does not, and is preferable in terms of avoiding the toxicity and costs associated with solvent recovery. Studies on melt-electrospinning have examined the effects of different heating technologies, such as electric current [3,4,5,6] or carbon dioxide (CO_2_) lasers [7,8,9,10,11,12,13,14,15].

We have demonstrated the preparation of nano- or submicron-fibers through laser-electrospinning (LES), which is a melt-electrospinning technique using CO_2_ laser irradiation for heating [10,11,12,13,14,15]. Uniform and ultra-fine nylon 6 and poly(L-lactide-co-ε-caprolactone) fibers, with an average diameter of approximately 1 μm and coefficient of variation (CV) of less than 20% [10], were successfully obtained.

Poly(ethylene terephthalate) (PET) has been widely used to create fibers, films, and bottles, and has myriad other applications. Applying the monofilament winding system to the LES, we have also investigated the influences of the drawdown ratio, laser power, and applied voltage on the structure and properties of the laser electro-spun PET fibers with diameters of approximately 12–18 μm [11]. When the drawdown ratio was increased, the birefringence and boil-off shrinkage of the electro-spun PET fibers increased, while their natural draw ratio and elongation at break decreased. In addition, when either the laser power or the applied voltage was increased, the birefringence decreased, whereas the natural draw ratio and elongation at break increased.

Ogata et al. also demonstrated the preparation of PET fibers with diameters of approximately 1 μm using a melt-electrospinning system equipped with a CO_2_-laser melting device. Rod-like PET samples with different intrinsic viscosities (IV) were supplied for the production of webs [8]. They reported that the diameter of the resultant PET fibers decreased as laser power increased, while the obtained minimum diameters were scarcely influenced by the IV. In addition, the electro-spun PET fibers did not show any crystalline orientation in the wide-angle X-ray diffraction (WAXD) measurement. Excluding the paper by Ogata [8], we could not find any other paper that discussed the preparation of PET webs via LES.

In this current research, attempts were made to prepare PET webs consisting of finer fibers through LES and subsequent annealing processes. The structure and properties of the obtained webs were evaluated. Similar analyses were performed for the as-received and annealed, unoriented amorphous PET films for comparison. To the best of our knowledge, this is the first attempt to analyze molecular orientation in PET fibers prepared by LES.

## 2. Materials and Methods

### 2.1. Preparation of Fiber Webs

#### 2.1.1. Materials

PET pellets (UNITIKA Ltd., Osaka, Japan, MA-2103, intrinsic viscosity 0.68 dL/g) were used in this study. The polymer contained a small amount of TiO_2_. After drying in a vacuum at 100 °C for 6 h and then at 120 °C for 6 h, the PET pellets were fed to an extrusion system, consisting of an extruder and a metering pump, and extruded from a spinneret with a single hole 1.0 mm in diameter at a throughput rate of 4.7 g/min and extrusion temperature of 290 °C. The extruded filament was taken up at a velocity of 200 m/min using a winder placed approximately 3.3 m below the spinneret. The prepared PET fibers [fiber diameter: 151 ± 8 μm (CV: 5%)] were then supplied to the LES process.

#### 2.1.2. Laser-Heated Electrospinning and Annealing Processes

**Figure 1:**
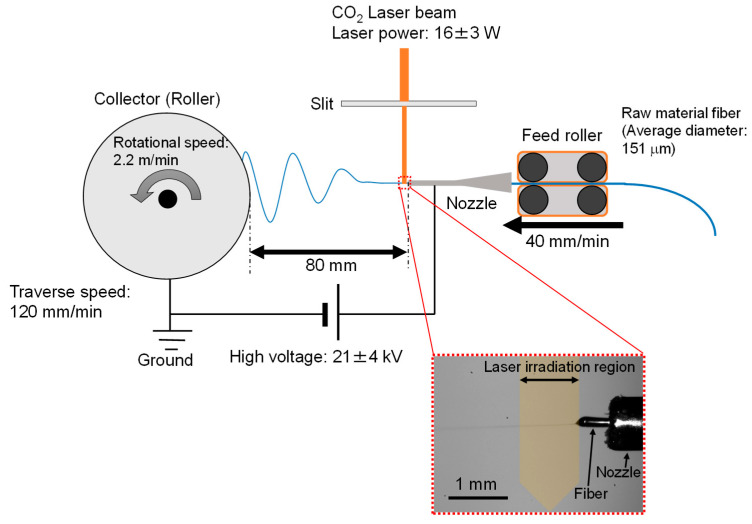
Schematic of the laser-electrospinning (LES) system.

The LES system (NEU-010, Katotech Co., Ltd., Kyoto, Japan) with a CO_2_ laser light source (PIN-30R, Onizca Glass Co., Ltd., Oume, Japan) is schematically shown in Figure 1. A PET fiber of approximately 150 μm in diameter prepared by the melt-spinning process was fed to the LES system from a tube nozzle at a rate of 40 mm/min using a feed roller. The nozzle had an outer diameter of 0.91 mm and an inner diameter of 0.60 mm (20 gauge). A high voltage of 21 ± 4 kV was applied to the nozzle. The PET fiber protruding from the nozzle was heated with the irradiation of a CO_2_ laser (wavelength of 10.6 μm, power of 16 ± 3 W) and driven to the collector by the high voltage. The collector roller was positioned 80 mm away from the tip of the nozzle, while the laser beam axis was positioned approximately 1 mm away from the nozzle tip. The laser beam was converged using a cylindrical lens and cutoff with an alumina slit to an elliptically shaped beam with short and long axes of approximately 1 and 4 mm, respectively, where the long axis was perpendicular to the direction of fiber running.

The relative humidity of the atmosphere was maintained below 40% using a dry-air flow at a room temperature of approximately 25 °C. The thinning behavior of the fiber in the process was observed using a charge-coupled device (CCD) camera equipped with a telecentric lens with 2× magnification and a personal computer (Panasonic Corp., Kadoma, Japan) with the software WinROOF 2015 version 3.0.0 (MITANI Corp., Fukui, Japan). Based on the in-situ observation of the thinning behavior of the protruded fiber, the laser power and the applied voltage were adjusted so as to stabilize the spin-line near the nozzle.

A PET web was formed on the collector roller with a diameter and width of 100 mm at a rotational surface speed of 2.2 m/min. For the homogenization of the web thickness, linear reciprocating motion along the rotation axis was additionally applied to the collector with a traverse range and speed of 50 mm and 120 mm/min, respectively. After the formation of the PET web by the LES process, a web sample with the dimensions of 50 mm × 50 mm with an area density of approximately 5 mg/m^2^ was placed on an amorphous PET film of the same size to prevent sag and detachment from the chuck due to the weight of the web. The thickness of the film was 246 ± 3 μm (CV: 1%). The web and film were adhered using double-sided adhesive tape with a width of 5 mm around the outer rim of the film. Annealing of some samples was carried out using a dry-air oven under the fixed-size condition. The temperature was increased at a rate of approximately 3 °C/min and maintained at 116 °C or 150 °C for 5 min. Along with the characterization of the prepared webs, an analysis of the amorphous PET film was conducted for comparison.

### 2.2. Characterization

#### 2.2.1. Scanning Electron Microscopy

The electrospun fiber webs were coated with Au by ion sputtering (E-1010, HITACHI Co., Ltd., Tokyo, Japan) and observed using a scanning electron microscope (SEM; VE-7800, KEYENCE Co., Ltd., Osaka, Japan). For each prepared PET web, the diameters of 100 fibers were measured from the SEM images using an image analysis software (Image J version 1.52v), and the average and coefficient of variation (CV) were evaluated.

#### 2.2.2. Polarizing Microscopy

The diameter and optical retardation of the raw material and electro-spun fibers were measured using a polarizing microscope (BX53-P, Olympus Co., Ltd., Tokyo, Japan) equipped with a polarizing filter and a Berek or Senarmont compensator. Birefringence, Δ*n*, was calculated using Equation (1):

(1) $$\Delta n\;=\;\frac{R}{d}$$

where *d* is the fiber diameter and *R* is the optical retardation.

#### 2.2.3. Wide-Angle X-ray Diffraction

WAXD 2-D patterns and intensity distribution profiles of the PET web and film samples were obtained using an X-ray generator (Rigaku Co., Akishima, Japan) and a CCD detector. The WAXD analysis was performed by generating the X-ray beam at a voltage of 45 kV and a current of 60 mA. A Ni-filtered CuKα beam was used.

#### 2.2.4. Differential Scanning Calorimetry

For the PET web and film samples, differential scanning calorimetry (DSC) analysis was performed (Q100 MI analyzer, TA Instruments Co, Inc., New Castle, DE, USA). The samples were measured within a temperature range of 25 °C to 300 °C at a heating rate of 10 K/min. Approximately 1 mg and 3 mg of the web and film samples, respectively, were weighed for measurement. Dry nitrogen was used as the purge gas at a flow rate of 50 mL/min. The DSC measurements were also conducted for the raw material fiber sample (sample mass of approximately 3 mg), which was cut into a powder-like form. The crystallinity of the samples was analyzed from the crystal melting endotherm, which was obtained by subtracting the exothermic heat of cold crystallization from the endothermic heat of melting. A heat of fusion of 140.1 J/g for the 100% crystallinity was employed for the calculation of crystallinity [16].

## 3. Results and Discussion

### 3.1. Fiber Diameters

**Figure 2:**
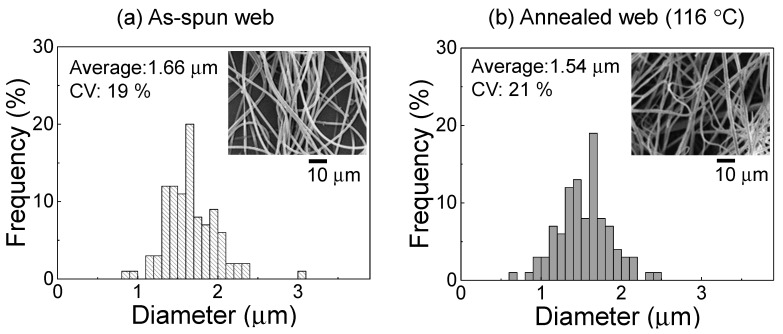
Fiber diameter distribution in the (**a**) as-spun poly(ethylene terephthalate) (PET) web and (**b**) web after annealing at 116 °C for 5 min. The average value and coefficient of variation of the fiber diameters, as well as the SEM image of the web, are shown in the insets.

The fiber diameter distributions of the PET web samples before and after annealing at 116 °C are shown in Figure 2 along with the corresponding SEM images. The average and CV values obtained from the diameter distribution are also shown. We proved that the PET web with ultra-fine fibers and fairly uniform fiber diameter (average fiber diameter of 1.66 μm and a CV of 19%) was obtained by the LES process. There was no practical change in fiber diameter after annealing at 116 °C, indicating that the length of the fibers in the web did not change during the annealing process because of the fixation of the web size.

### 3.2. Birefringence

**Figure 3:**
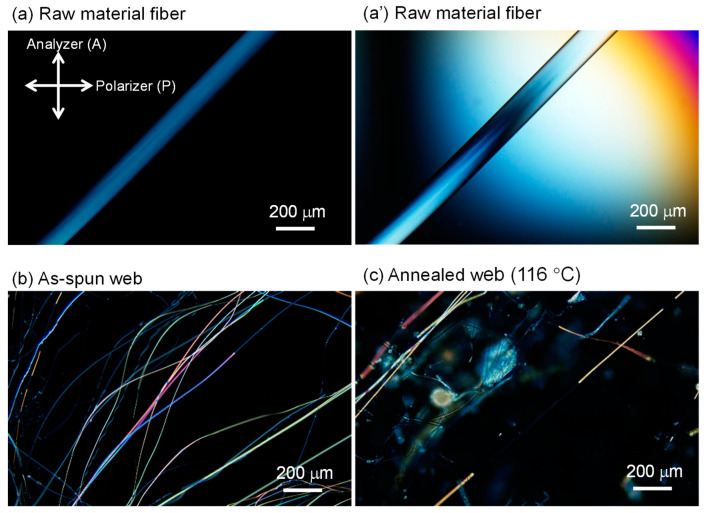
Micrographs observed under a polarizing microscope with cross-polarization for the (**a**), (**a’**) raw material fiber (melt-spun fiber supplied for LES), (**b**) as-spun web, and (**c**) web after annealing at 116 °C for 5 min. The directions of polarization for a polarizer and an analyzer are indicated in the figure. A Berek compensator was inserted in (**a’**).

**Figure 4:**
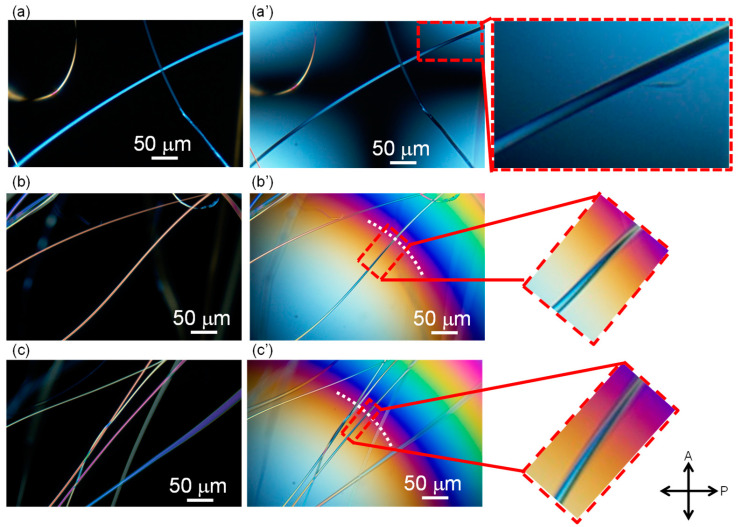
Typical examples of fibers in the as-spun web observed under a polarizing microscope under (**a**–**c**) cross-polarization and (**a’**–**c’**) cross-polarization with the insertion of a Berek compensator. Images shown with (**a’**) no addition of retardation at the center of the image and (**b’**), (**c’**) a position with the addition of retardation of approximately 550 nm is indicated with a white dotted line.

**Figure 5:**
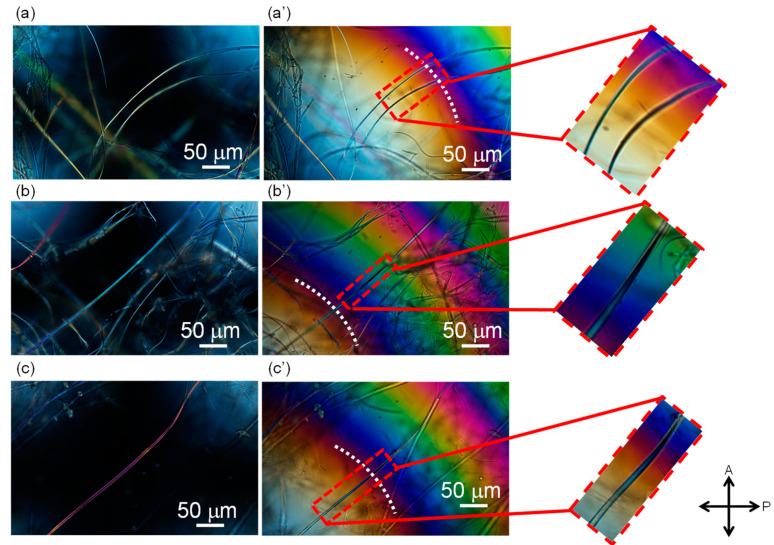
Typical examples of fibers in the web after annealing at 116 °C for 5 min, observed under a polarizing microscope with the insertion of a Berek compensator and (**a**–**c**) no addition of retardation at the center of the image and (**a’**–**c’**) a position with the addition of retardation of approximately 550 nm is indicated with a white dotted line.

Micrographs of the PET fibers observed under a polarizing microscope with cross-polarization are shown in Figure 3, Figure 4 and Figure 5. Some images were taken by inserting the Berek compensator (Olympus Co., Ltd., Tokyo, Japan) with an applied mean optical retardation of approximately 0 and 550 nm. From these images, the diameter, retardation, and birefringence of various fibers were evaluated, as shown in Table 1.

The birefringence of the raw material fibers (melt-spun fibers fed for LES) was low, as expected. On the other hand, the LES fibers in the webs showed a bimodal distribution of low and high birefringence. The diameters of the fibers were rather uniform in comparison with the wide distribution of the birefringence. Due to the limited number of measurements, a clear correlation between the birefringence and fiber diameter could not be identified.

The lower birefringence values (10.5 and 16.3 × 10^−3^) and the higher birefringence values (106 and 121 × 10^−3^) are comparable to those of melt-spun fibers prepared at spinning speeds of 2–3 km/min and 6–7 km/min, respectively [17]. In other words, the lower and higher birefringence values correspond to the degrees of orientation for undrawn yarn (UDY) and highly oriented yarn (HOY), respectively. After annealing the web at 116 °C, the fibers with high and low birefringence still co-existed, and the high birefringence part showed a significant increase in birefringence. This result is in accordance with the changes in birefringence of high-speed spun fibers after annealing.

### 3.3. WAXD Analysis of the Web

**Figure 6:**
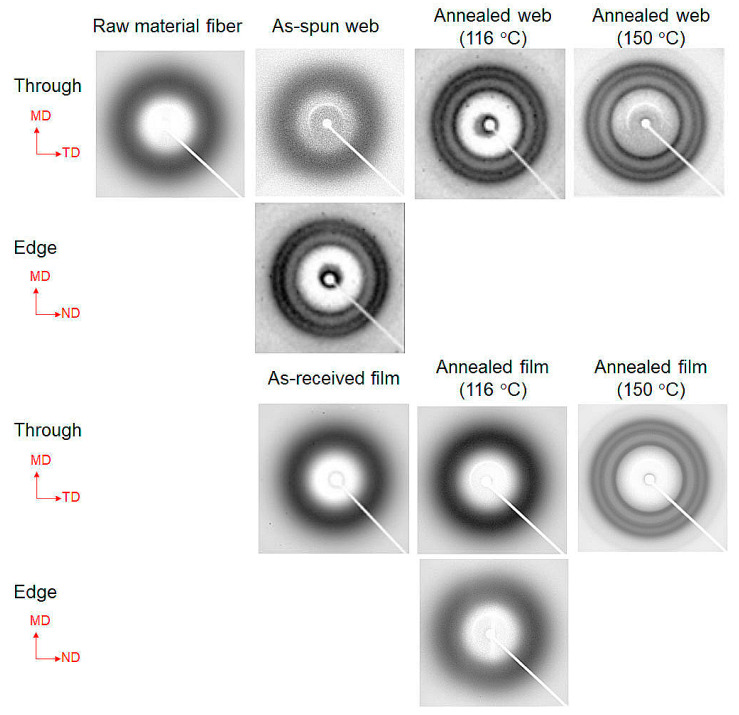
Wide-angle X-ray diffraction (WAXD) patterns of the web and film samples of PET obtained from the through- and edge-direction. The machine direction (MD), transverse direction (TD), and normal direction (ND) are indicated in the figure.

**Figure 7:**
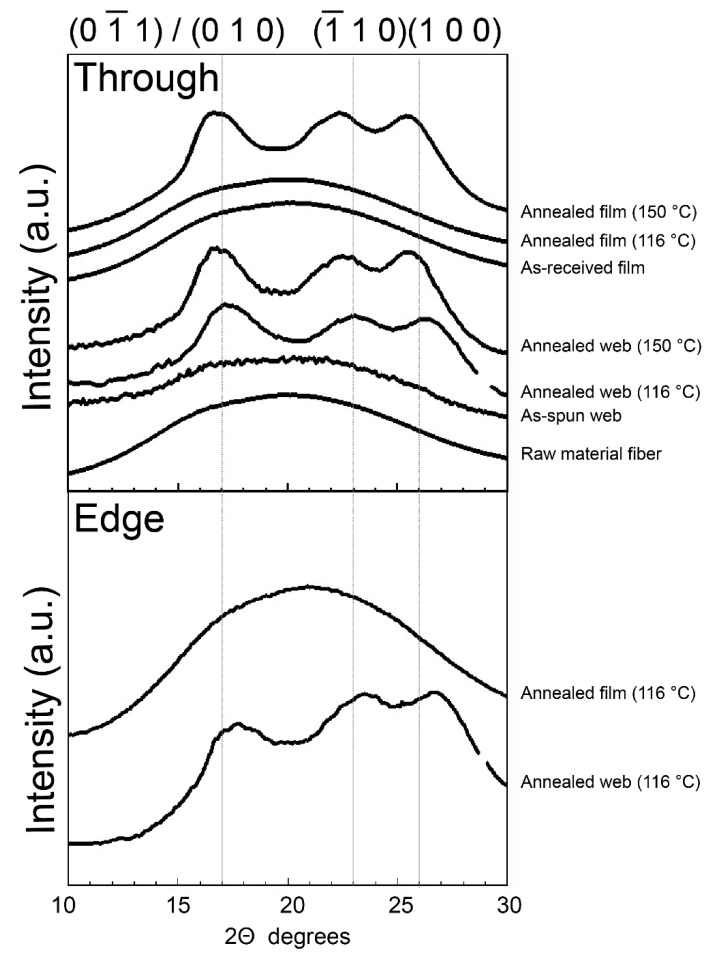
WAXD intensity profiles of the web and film samples obtained by averaging the intensities along the azimuthal direction from 0 to 180°.

The WAXD patterns obtained from the through- and edge-directions of the web and film samples of PET are shown in Figure 6. In this figure, the MD, TD, and ND correspond to the winding/machine direction, transverse direction, and normal direction, respectively. The WAXD intensity profiles of the web and film samples obtained by averaging the intensity along the azimuthal direction from 0° to 180° are shown in Figure 7. The intensity peaks at approximately 2θ = 17°, 23°, and 26° correspond to the $(0\bar{1}1$)/(010), $(\bar{1}10$), and (100) reflections, respectively [18]. The appearance of only an amorphous halo was confirmed for all the samples (as-received film, raw material fiber for LES, and as-spun web) before annealing. After annealing at 116 °C for 5 min, the film samples did not exhibit crystalline reflections, whereas distinct crystalline reflections were observed for the annealed web samples. At an annealing temperature of 150 °C, the appearance of crystalline reflections was confirmed for both the film and fiber samples.

**Figure 8:**
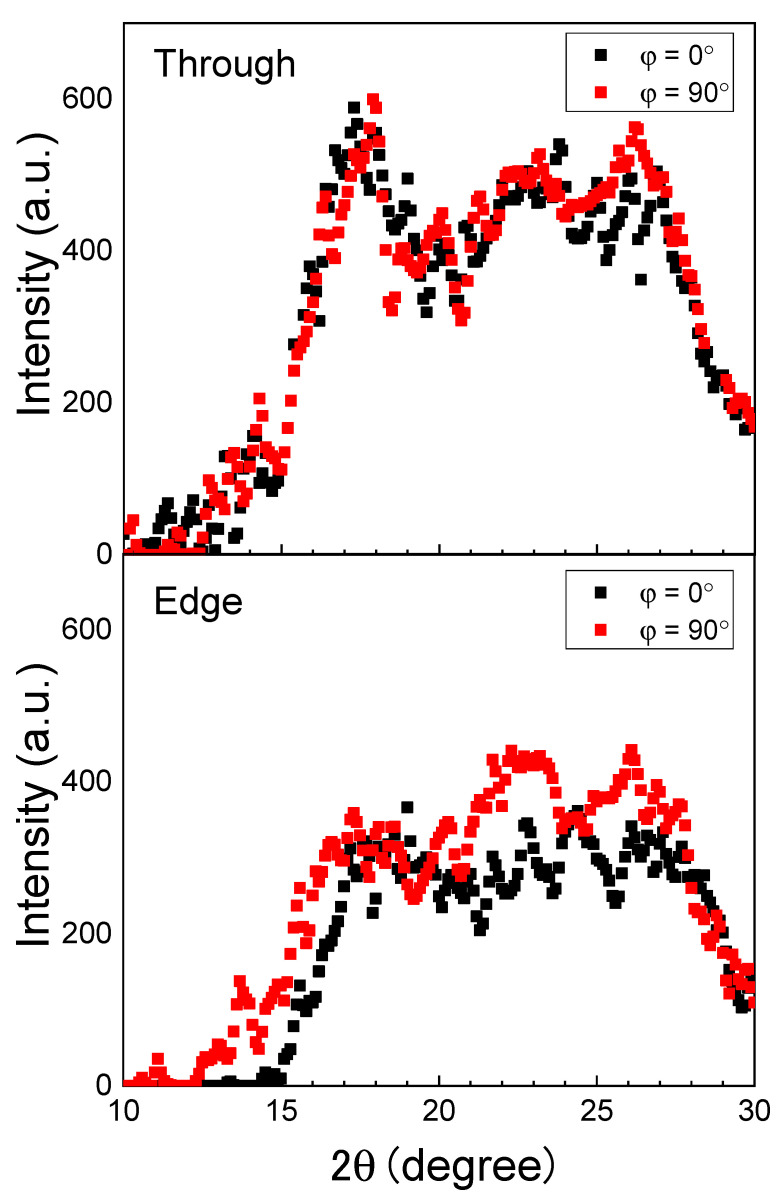
WAXD intensity profiles at the azimuthal angles, φ, of 0° and 90° for the web sample annealed at 116 °C.

For the web sample annealed at 116 °C, intensity profiles at azimuthal angles of 0° and 90° were prepared from the results of the through- and edge-direction WAXD measurements. As can be seen in Figure 8, the crystalline peak intensities were slightly stronger at 90° than at 0° for the edge-view measurement, although no clear difference was found for the through-view measurement. This result suggests the existence of a certain degree of c-axis orientation of the crystals in the web fibers, where the fibers were aligned parallel to the web surface.

### 3.4. DSC Analysis

**Figure 9:**
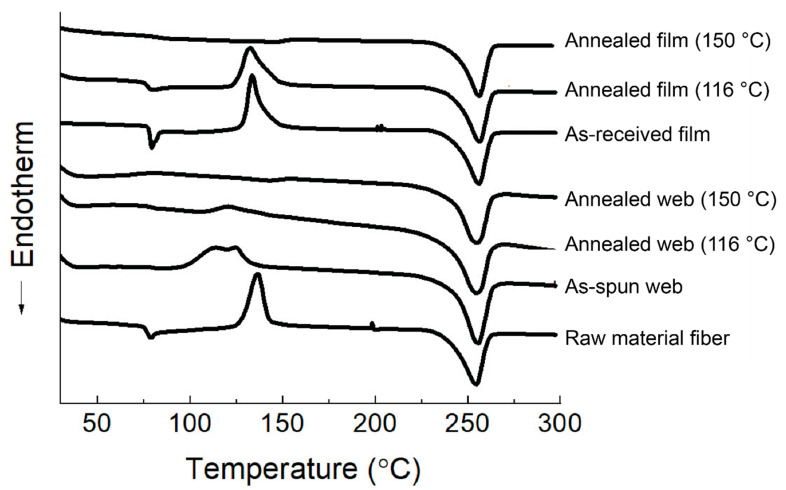
Differential scanning calorimetry (DSC) thermograms of the web and film samples.

**Figure 10:**
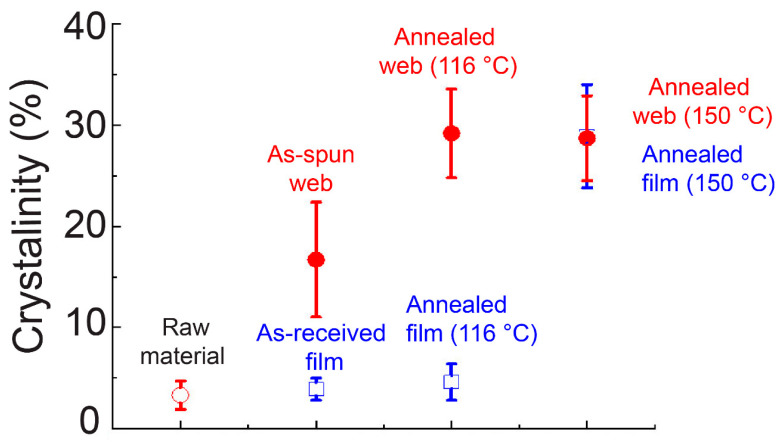
Crystallinity analyzed from DSC thermograms of web and film samples.

The DSC thermograms of the web and film samples are shown in Figure 9. The crystallinity obtained from Figure 9 is shown in Figure 10. Glass transition was clearly observed at approximately 77 °C for the as-received film and the raw material fiber, whereas that of the as-spun web was obscure. After annealing at 150 °C, glass transition could not be observed both for the web and film samples. On the contrary, after annealing at 116 °C, the film sample still showed a distinct glass transition, which was not observed for the web sample. In addition, an exothermic peak of cold crystallization for the as-received film and raw material fiber appeared at approximately 131 °C and 138 °C, respectively, whereas that of the as-spun web became broad and appeared at a lower temperature of approximately 118 °C, indicating the existence of fibers with a certain degree of molecular orientation. The broad cold crystallization peak may suggest the existence of an orientation distribution of the fibers with relatively low birefringence. After annealing at 116 °C, the web showed only a slight trace of the cold crystallization peak, whereas the film annealed at 116 °C showed a distinct cold crystallization peak at approximately 130 °C. When the annealing temperature was raised to 150 °C, the cold crystallization peak disappeared completely for both the web and film samples.

These results suggest that the annealing period of 5 min at 116 °C was not sufficient for the crystallization of the film with virtually no orientation, while the web sample could crystallize under these conditions because a certain degree of orientation was imposed during LES. Therefore, after annealing at 116 °C, the crystallinity of the film was approximately 5%, while that of the web samples was approximately 29%, as shown in Figure 10. After annealing at 150 °C, both the web and film samples showed a crystallinity of approximately 29%.

Although no clear diffraction peak was confirmed in the WAXD results, the crystallinity of the as-spun web sample calculated from the DSC thermogram was approximately 17%, which was higher than those of the as-received film and the raw material fiber for LES (~3%). The cold crystallization temperature of the as-spun web corresponded to that of the high-speed spun fibers prepared at the take-up velocity of 2–3 km/min. On the other hand, the crystallinity of the as-spun web analyzed from the DSC thermogram corresponded to that of the high-speed spun fibers prepared at the take-up velocity of 3–4 km/min [17]. The discrepancy regarding the relationship between the cold crystallization temperature and crystallinity from the DSC measurements for the LES web and the high-speed spun fibers can be explained as follows. The birefringence measurements of the fibers in the web confirmed the co-existence of fibers with low and high birefringence, as shown in Table 1. It can be speculated that the crystallinity higher than that expected from the cold crystallization temperature was obtained because of the presence of highly oriented fibers, which did not show any cold crystallization peak. It should be noted that such high-birefringence fibers did not exhibit a crystalline peak in the WAXD analysis, as shown in Figure 6.

## 4. Conclusions

PET fiber webs were prepared through laser-heated electrospinning (LES) and subsequent annealing processes. Through the analyses of the structure and properties of the obtained webs, it was proved that the PET web with ultra-fine fibers of fairly uniform fiber diameter (average fiber diameter of 1.66 μm and its CV of 19%) was obtained by the LES process. Birefringence of the LES fibers in the web showed a bimodal distribution of low and high values. After the annealing of the web at 116 °C for 5 min, the fibers with high and low birefringence still co-existed, where the high birefringence part showed a significant increase in birefringence. In the wide-angle X-ray diffraction (WAXD) analysis, the appearance of only an amorphous halo was confirmed for the as-spun web sample before annealing, even though the existence of the fibers of high birefringence was confirmed. After the annealing of the web samples at 116 °C or 150 °C for 5 min, distinct crystalline reflections were observed. In terms of the crystallinity of the as-spun web samples, although crystalline diffraction peaks were not observed in the WAXD measurements, the crystallinity estimated from the DSC thermogram showed a relatively high value of about 17%. There was a discrepancy between the results for LES web and high-speed spun fibers if the relationship between the cold crystallization temperature and the crystallinity from DSC was considered, in that higher crystallinity was observed for the web in comparison with its relatively low cold crystallization temperature. The presence of highly oriented fibers, which might not show any cold crystallization peak, was speculated to be the origin of this discrepancy. After annealing at 116 °C or 150 °C, the crystallinity of the web samples reached about 29%.

## Figures and Tables

**Table 1:** Retardation, *R*, diameter, *d*, and birefringence, Δ*n*, of various fibers analyzed using a polarizing microscope.

Sample	*R* (nm)	*d* (μm)	Δ*n* × 1000
Raw material fiber	139.5	152.2	0.9
	133.4	59.7	2.2
As-spun web	438.5	3.6	121
	64.2	4.0	16.3
	310.1	2.9	106
	24.1	2.3	10.5
Annealed web (116 °C)	336.0	2.2	150
	30.3	5.2	5.8
	758.0	3.7	203
	555.0	3.7	149
	804.0	5.2	154
	267.0	2.2	119
